# Oleuropein, the Main Polyphenol of *Olea europaea* Leaf Extract, Has an Anti-Cancer Effect on Human BRAF Melanoma Cells and Potentiates the Cytotoxicity of Current Chemotherapies

**DOI:** 10.3390/nu10121950

**Published:** 2018-12-08

**Authors:** Jessica Ruzzolini, Silvia Peppicelli, Elena Andreucci, Francesca Bianchini, Arianna Scardigli, Annalisa Romani, Giancarlo la Marca, Chiara Nediani, Lido Calorini

**Affiliations:** 1 Department of Experimental and Clinical Biomedical Sciences “Mario Serio”, University of Florence, 50134 Florence, Italy; jessica.ruzzolini@unifi.it (J.R.); silvia.peppicelli@unifi.it (S.P.); e.andreucci@unifi.it (E.A.); francesca.bianchini@unifi.it (F.B.); 2 PHYTOLAB (Pharmaceutical, Cosmetic, Food Supplement Technology and Analysis)-DiSIA, Scientific and Technological Pole, University of Florence, 50134 Florence, Italy; arianna.scardigli@unifi.it (A.S.); annalisa.romani@unifi.it (A.R.); 3 Mass Spectrometry Laboratory, Clinic of Pediatric Neurology, Meyer University Children’s Hospital, 50139 Florence, Italy; giancarlo.lamarca@unifi.it; 4 Istituto Toscano Tumori and Center of Excellence for Research, Transfer and High Education DenoTHE University of Florence, 50139 Florence, Italy

**Keywords:** BRAF melanoma, chemotherapeutics, extra virgin oil, Oleuropein, olive leaf extract

## Abstract

Oleuropein (Ole), a secoiridoid glucoside present in *Olea europaea* leaves, gained scientific interest thanks to its several biological properties, including the anticancer one. We verified whether Ole might potentiate the cytotoxicity of conventional drugs used to treat melanoma, disclosing a potentially new therapeutic strategy. We tested the cytotoxic action of Ole alone or in combination with chemotherapeutics on A375 human melanoma cells. We found that Ole was able, at a dose of 500 µM, to stimulate apoptosis, while at a non-toxic dose of 250 µM, it affected cell proliferation and induced the downregulation of the pAKT/pS6 pathway. A dose of 250 µM Ole did not potentiate the effect of Vemurafenib (PLX4032), but it succeeded in increasing the cytotoxic effect of Dacarbazine (DTIC). The major effect was found in the association between Ole and Everolimus (RAD001), also on PLX4032-resistant BRAF melanoma cells, which possibly cooperate in the inhibition of the pAKT/pS6 pathway. Of interest, an olive leaf extract enriched in equimolar Ole was more effective and able to further improve DTIC and RAD001 efficacy on BRAF melanoma cells with respect to Ole alone. Therefore, Ole represents a natural product able to potentiate a wide array of chemotherapeutics against BRAF melanoma cells affecting the pAKT/pS6 pathway.

## 1. Introduction

Melanoma originates in the skin and although it represents one of the rarer forms of skin cancer, it underlies the majority of skin cancer-related deaths [1] whose incidence has more than doubled in the last 10 years. About 50–60% of melanomas show the BRAF^V600E^ mutation [2] where a valine is replaced by either an arginine (V600K) or glutamic acid (V600E). This mutation implies the constitutive activation of extracellular signal-regulated kinases (ERK) signaling that leads to the increase of proliferation and transformation [2,3,4,5,6]. Although this mutation commonly occurs in melanoma, it should be noted that the mutation itself is not sufficient to cause cancer since it is also found in benign melanocytic lesions [1,7,8]. Likewise, the phosphoinositol-3-kinase (PI3K)/protein kinase B (AKT)/mammalian target of the rapamycin (mTOR) pathway is also involved in melanoma genesis, and its activation often leads to increased cell survival, proliferation, and motility [9]. Current protocol for melanoma treatment is dependent on the condition of the tumor at the time of disease detection; if diagnosed early, melanoma may be removed using surgery, but if it spreads to the lymph nodes, surgery will be more invasive and chemo- and immunotherapy will be associated with the treatment. Unfortunately, these therapies may undergo patient resistance and generate host tissue damage. In particular, intrinsic resistance to apoptosis of melanoma cells is one of the main causes of anticancer therapy failure. Until recently, the prognosis for advanced malignant melanoma was poor, but the discovery of the major pathways involved in melanoma progression and resistance prompted the use of new therapeutic agents.

Therefore, new strategies targeting melanoma cells, which also reduce resistance and patient side effects, need to be developed and a combination of conventional treatment with biological agents (the so-called complementary therapy) might be an important breakthrough.

The Mediterranean diet is considered an important preventive instrument against chronic diseases, such as neurodegenerative and cardiovascular ones, and cancer [10,11,12]. In particular, epidemiological studies indicate that the dietary consumption of extra virgin olive oil (EVOO) has a protective effect in Mediterranean populations [13,14,15,16]. EVOO is a functional food with a high level of monounsaturated fatty acids and a minor level of highly bioactive multiple components, including polyphenols, to which have been mainly attributed the beneficial effects [17,18,19]. The phenol composition of olive oil includes the phenolic alcohols, hydroxytyrosol (HT, 3,4dihydroxyphenylethanol, 3,4-DHPEA, DOPET), and tyrosol (p-hydroxyphenylethanol, p-HPEA) together with their secoiridoid precursors. The main secoiridoid in olive oil is 3,4-dihydroxyphenylethanol-elenolic acid (3,4-DHPEA-EA), whose glycated form is also known as Oleuropein (Ole), the main agent responsible for the bitter taste of olive leaves and drupes. Ole has been reported to have many pharmacological properties, among which are antioxidant, anti-inflammatory, cardioprotective, neuroprotective, and hepatoprotective properties [20,21]. Recently, the accumulating in vitro and in vivo experiments, together with epidemiological and clinical data, have provided support to the anti-tumor properties of Ole toward different tumor histotypes, such as breast, colon, and lung cancer [22,23]. Of translational importance is that Ole was found to be a powerful sensitizer of the Doxorubicin-mediated killing of prostate and breast cancer cells [24,25]. In fact, it lowers the cytotoxic dose of Doxorubicin, while producing an anti-proliferative effect in cancer cells.

The aim of our work is to verify both the Ole cytotoxic effect and its cooperation with the current drugs used in BRAF melanoma. We have found that Ole promotes cytotoxicity of Dacarbazine (DTIC), a guanine methylating agent, whose treatment, was approved by the Food and Drug Administration (FDA) and of the mTOR inhibitor Everolimus (RAD001), and that its combination with RAD001 was also an effective strategy in treating Vemurafenib (PLX4032)-resistant BRAF melanoma cells, where Vemurafenib (PLX4032) is a BRAF inhibitor. Overall, these findings disclose the wide possibility of using Ole in the complementary therapy of melanoma.

## 2. Materials and Methods

### 2.1. Cell Lines and Culture Conditions

In this study, we used A375 human melanoma cell lines, obtained from American Type Culture Collection (ATCC, Rockville, MD, USA). In some experiments we used also the human melanoma cell lines WM266-4 (from ATCC) and M21 (kindly provided by Dr. Antony Montgomery, The Scripps Research Institute, La Jolla, CA, USA). Melanoma cells were cultivated in Dulbecco’s Modified Eagle Medium high glucose (DMEM 4500, EuroClone, Milan, Italy) supplemented with 10% fetal bovine serum (FBS, Boerhinger Mannheim, Binger Strasse, Ingelheim am Rhein, Germany), at 37 °C in a humidified atmosphere containing 90% air and 10% CO_2_. Viability of the cells was determined using a trypan blue exclusion test. Cultures were periodically monitored for mycoplasma contamination using Chen’s fluorochrome test [26].

A375 melanoma cells resistant to PLX4032 were kindly provided by Laura Poliseno from University of Pisa and they were obtained as explained in Reference [27]. PLX4032-resistant A375 melanoma cells were maintained without PLX4032 overnight before the start of the experiment.

According to the experiments, cells were treated with Oleuropein glucoside (purity ≥ 90%) (Extrasynthese S.A., Lyon, Nord-Genay, France), DTIC (Sigma Aldrich, Milan, Italy), RAD001 (MedChem Express, Stockholm, Sweden) or PLX4032 (MedChem Express, Stockholm, Sweden).

### 2.2. MTT Assay

Cell viability was assessed using MTT (3-(4,5-dimethylthiazol-2-yl)-2,5-diphenyltetrazolium bromide) tetrazolium reduction assay (Sigma Aldrich, Milan, Italy). Cells were plated into 96-multiwell plates in complete medium without red phenol. The treatment was added to the medium culture at different doses and times, according to the experiment. Then the MTT reagent was added to the medium and plates were incubated at 37 °C. After 2 h, MTT was removed and the blue MTT-formazan product was solubilized with dimethyl sulfoxide (DMSO) (Sigma Aldrich, Milan, Italy). The absorbance of the formazan solution was read at 595 nm using the microplate reader (Bio-Rad Via Cellini, Segrate (Milan), Italy).

### 2.3. Sample Preparation for Mass Spectrometry Analysis

Cells were washed with ice-cold phosphate buffered saline (PBS) containing 1 mM Na4VO3, scraped in PBS, centrifuged for 5 min at 1200 rpm and lysed with ice-cold water. Cells were sonicated three times for 5 min and supernatants were collected for mass spectrometry analysis.

The samples were measured using analytical High Performance Liquid Chromatography (HPLC) coupled to API 4000 (AB SCIEX, Toronto, ON, Canada) equipped with the TurboIonSpray source operated in negative ion mode, as previously described [28] with slight modifications. Briefly, the capillary voltage of the mass spectrometer was set to −4500 V, the “turbo” gas flow was 10 L/min of air heated at 400 °C. The following transitions were monitored in MRM mode (multiple reaction monitoring): *m*/*z* 153.1 > 123.1 for HT; 377.4 > 307.3 for oleuropein aglycone; 539.5 > 275.3 for Ole. Optimal CE (collision energy) and CXP (collision cell exit potential) were found at −18 V and −8 V for HT; −16 V and −6 V for both Oleuropein aglycone and Ole, respectively. The resulting DP (declustering potential) was −70 V. The chromatographic experiments were undertaken by using a Series 1290 Infinity LC System (Agilent Technologies, Waldbronn, Germany) HPLC Capillary Pump coupled to an Agilent Micro ALS autosampler, both being fully controlled from the API 4000 data system. Liquid chromatography was performed using a Zorbax eclipse C18 3 × 150 mm, 3.5 µm HPLC column (Agilent Techonologies, Waldbronn, Germany). Column flow was 0.4 mL/min using a water/acetonitrile (50:50) and 0.05% formic acid in an isocratic elution system. The eluent from the column was directed to the TurboIonSpray probe without a split ratio.

### 2.4. Evaluation of Apoptosis

Apoptosis was measured using flow cytometry, using the Annexin V staining. Cells were washed once with PBS, detached with Accutase (Euroclone, Milan, Italy), resuspended in 100 mL of 1× Annexin-binding buffer at the concentration of 1 × 10^6^ cells/mL, stained with 5 mL of Annexin V FITC-conjugated (ImmunoTools, Friesoythe, Germany) and 1 mL of 100 mg/mL propidium iodide (PI) working solution and incubated at 4 °C in the dark condition for 15 min. Then, 400 mL of 1× Annexin Binding Buffer was added to each sample and cells were analyzed using flow cytometry (BD-FACS Canto) to find out the viability (annexin V and PI negative, Q3), early apoptosis (annexin V positive and PI negative, Q4), or late apoptosis (annexin V and PI positive, Q2). A minimum of 10,000 events were collected, as previously described [26].

### 2.5. Cell Cycle Analysis

Cell cycle distribution was analyzed via the DNA content using the PI staining method. Cells were centrifugated and stained with a mixture of 50 µg/mL PI (Sigma-Aldrich, St. Louis, MO, USA), 0.1% trisodium citrate and 0.1% NP40 (or triton x-100) (Sigma-Aldrich, St. Louis, MO, USA) in the dark at 4 °C for 30 min. The stained cells were analyzed via flow cytometry (BD-FACS Canto, BD Biosciences, Franklin Lakes, NJ, USA) using red propidium-DNA fluorescence [26].

### 2.6. Invasion Assay

Cells invasion was performed using a polycarbonate cell culture insert with a pore size of 8.0 µm (Sigma-Aldrich) coated with Matrigel (0.25 µg/µL; BD Biosciences, Franklin Lakes, NJ, USA). Cells suspended in 200 µL of their own growth medium were seeded in the upper compartment, while in the lower chamber, fresh complete medium was added as chemo attractant.

Cells were incubated for 6 h at 37 °C, 10% CO_2_ in air, and 25 µM Ilomastat was used as a control for metalloprotease inhibition (Millipore, Billerica, MA, USA). After incubation, filters were removed and the non-invading cells on the upper surface were wiped off mechanically with a cotton swab. Cells on the lower side of the filters were fixed overnight in ice-cold methanol, then stained using a DiffQuick kit (BD Biosciences, Franklin Lakes, NJ, USA) and pictures of randomly chosen fields were taken, as previously reported [26].

### 2.7. Plate Colony Forming Assay

Approximately 100 cells/mL surviving the different treatments were selected using the trypan blue exclusion test, seeded in fresh medium, and incubated for 10 days at 37 °C. Cells were washed with PBS, fixed in cold methanol, and stained using a Diff Quik kit (BD Biosciences). The stained colonies were photographed with a digital camera and the number of colonies in each well was counted.

### 2.8. Western Blotting Analysis

Cells were lysed and separated using electrophoresis as previously described [26]. Proteins were transferred to a polyvinylidene difluoride (PVDF) membrane and, subsequently, the membrane was probed at 4 °C overnight with primary antibodies diluted in a solution of 1:1 Odyssey blocking buffer (LI-COR^®^ Bioscience, Lincoln, NE, USA)/Tween (Sigma-Aldrich, St. Louis, MO, USA) -PBS buffer. The primary antibodies were: rabbit anti poly (ADP-ribose) polymerase (PARP)1 and cleaved PARP1 (1:1000, Cell signaling Technology, Danvers, MA, USA), rabbit anti-cleaved caspase 3 (1:1000, IDT, Tema Ricerca, Bologna, Italy), rabbit anti pAKT (1:1000, Cell signaling Technology, Danvers, MA, USA), rabbit anti AKT (1:1000, Cell signaling Technology, Danvers, MA, USA), rabbit anti pERK (1:1000, Cell signaling Technology, Danvers, MA, USA), and rabbit anti ERK (1:1000, Cell signaling Technology, Danvers, MA, USA). The membrane was washed in T-PBS buffer, incubated for 1 h at room temperature with goat anti-rabbit IgG Alexa Flour 750 antibody or with goat anti-mouse IgG Alexa Fluor 680 antibody (Invitrogen, Monza, Italy), and then visualized using an Odyssey Infrared Imaging System (LI-COR^®^ Bioscience, Lincoln, NE, USA). Mouse anti-β-Tubulin monoclonal antibody (1:1000, Cell signaling Technology, Danvers, MA, USA) was used to control for equal protein loading.

## 3. Olive Leaf Extract’s Preparation

### 3.1. Plant Material

*Olea europaea* L. (cultivar Moraiolo), organic green leaves, were collected in April 2018 in Tuscany (Vinci, Florence, Italy) and immediately processed.

### 3.2. Solvents and Reagents

All the solvents (HPLC grade) and formic acid (ACS reagent) were purchased from Aldrich Chemical Company Inc. (Milwaukee, WI, USA). Tyrosol, luteolin 7-O-glucoside, chlorogenic, and Ole were obtained from Extrasynthese S.A. (Lyon, Nord-Genay, France). The HPLC-grade water was obtained via double-distillation and purification with a Labconco Water Pro PS polishing station (Labconco Corporation, Kansas City, MI, USA) [29].

### 3.3. Extraction and Lyophylisation

The extraction using 15% of *Olea* leaves (45 g leaves/300 g double-distilled and purified water), was performed in water at a temperature of 50 °C for 60 min and at room temperature overnight (12 h) [29]. The final powder was obtained using lyophilization with the LYOVAC GT 2, with a freeze-drying yield of 1.85%.

### 3.4. Sample Preparation for Mass Spectrometry Analysis

The identity of the phenolic compounds of *Olea* dry extract powder and the composition of the solution used for the test in vitro, enriched in Oleuropein, was ascertained using data from the HPLC/DAD and HPLC/MS analyses, in accordance with a previous paper [30].

### 3.5. Statistical Analysis

Densitometric data are expressed as means ± standard errors of the mean (SEM) depicted using vertical bars from representative experiment of at least three independent experiments. Statistical analysis of the data was performed using ANOVA and Tukey’s multiple comparisons test, and *p* ≤ 0.05 was considered statistically significant.

## 4. Results

### 4.1. Ole Efficacy on BRAF Melanoma Cells

The effect of Ole on the cell viability was assessed on BRAF mutant (V600E) A375, WM266-4, and M21 melanoma cells following incubation for 72 h with an Ole concentration, ranging from 50 to 800 µM (corresponding to ≈25–400 µg/mL), using the MTT assay protocol. At 500–800 µM, Ole induced a very toxic effect that was able to almost totally reduce the viability of all melanoma cell lines (Figure S1a); at 250 µM (≈125 µg/mL), Ole caused a different but significant decrease of viability (about 30% in A375, 50% in WM266-4, and 0% in M21 vs. untreated cells). Starting from these results, for the later experiments we decided to use the A375 melanoma cell line, in which 250 µM Ole showed an intermediate toxic effect. In consideration of the possibility that the anticancer effects of Ole might be due to HT or its other metabolites [23,31,32], we performed some experiments to determine the presence of intracellular phenolic compounds after incubation from 15 min to 72 h with 250 µM Ole in A375 cells. As shown in Figure S1b, only Ole was detected in the cytoplasm using mass spectrometry analysis after only 15 min of incubation and was present without being metabolized until the 72nd hour of exposure (data not shown).

Prompted by the MTT assay results, we verified that by using a dose of 500 µM Ole, and not 250 µM, a significant percentage of A375 melanoma cells (about 90%) underwent apoptosis after 48 h, as demonstrated through the cytofluorimetric technique (Figure S2b). Based on these results, melanoma cells were exposed for 24–48 h to 250–500 µM Ole and the level of apoptosis, assessed uisng western blotting of PARP1, confirmed that only 500 µM Ole was able to promote the expression of a significant level of cleaved PARP1 after 48 h of treatment (Figure S2c). With the aim to verify whether Ole might potentiate drug efficiency on melanoma cells, we decided to use a non-toxic 250 µM dose, approximately corresponding to half maximal inhibitory concentration (IC50), previously determined in our laboratory (data not shown), which affected cell viability, evaluated using MTT (Figure S2a), without inducing cell apoptosis. On the other hand, 250 µM Ole reduced the cell proliferation rate of treated melanoma cells (Figure S2d) and inhibited pAKT/mTOR pathway, assessed by the decrease of densitometric analysis of AKT/S6 phosphorylation (Figure S2e).

Ole confirmed its anti-cancer role by also reducing cell invasive ability, one of the most important features of malignant phenotype, which is strongly linked with metastatic progression. A375 cells were treated with 250 µM Ole for 24 h, and evaluated for their ability to migrate through Matrigel in their own growth medium, and showed a reduced invasive activity with respect to the untreated cell (Figure S2f). These results suggested that Ole alone might be effective in treating BRAF melanoma cells, thus disclosing the possibility to analyze whether this polyphenol may also improve drug efficacy in advanced melanoma.

### 4.2. Combination of Ole with Vemurafenib (PLX4032)

**Figure 1:**
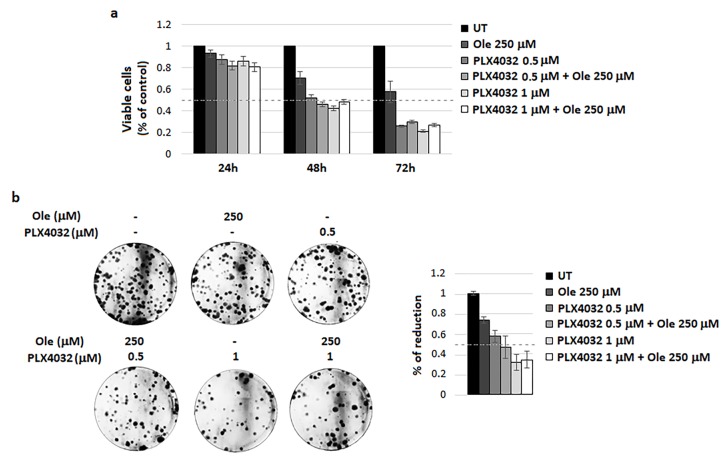
Ole-Vemurafenib (PLX4032) efficacy on A375 melanoma cells. (**a**) Dose–time response evaluated via MTT assay. (**b**) (**Left**) Colony forming units (CFU) assay of alive cells selected using a trypan blue exclusion test after 250 µM (≈125 µg/mL) Ole and/or d treatment for 72 h. (**Right**) Quantification data of the reduction of colony numbers compared to UT. UT = untreated.

First, we decided to investigate whether Ole might potentiate the activity of PLX4032, which is used for the targeted therapy of BRAF^V600E^ melanoma. Although this drug shows a very high clinical efficiency in metastatic melanoma patients harboring the BRAF V600E mutation, a wide type of BRAF melanoma tumors do not respond to PLX4032 or, in some cases, important clinical side effects limit the use of this drug, such as skin lesions and even cutaneous squamous cell carcinoma. Furthermore, due to the genetic heterogeneity of melanoma patients, the development of drug resistance represents a general phenomenon upon PLX4032 treatment. Several mechanisms of resistance to BRAF inhibitors have been proposed and most of them are based on pAKT/pS6 hyperactivation, the same pathway affected by Ole. Unfortunately, we did not find any potentiation of Ole on PLX4032 activity on A375 cells, as shown by the results obtained testing cell viability and efficiency to form colonies (Figure 1a,b)

### 4.3. Combination of Ole with Dacarbazine (DTIC)

DTIC belongs to the class of DNA-methylation agents and it is considered the main drug for the treatment of the advanced stage of melanoma. However, the benefit of the treatment with DTIC in 95% of cases is partial, with a 6-year median survival of 2%. Therefore, new therapeutic approaches need to be developed to improve chemotherapy efficacy and patient survival. However, treatment based on the combination of chemotherapy with other cytotoxic drugs has not resulted in response rates of durable remission, which were enough to increase the median survival of the patients [33].

**Figure 2:**
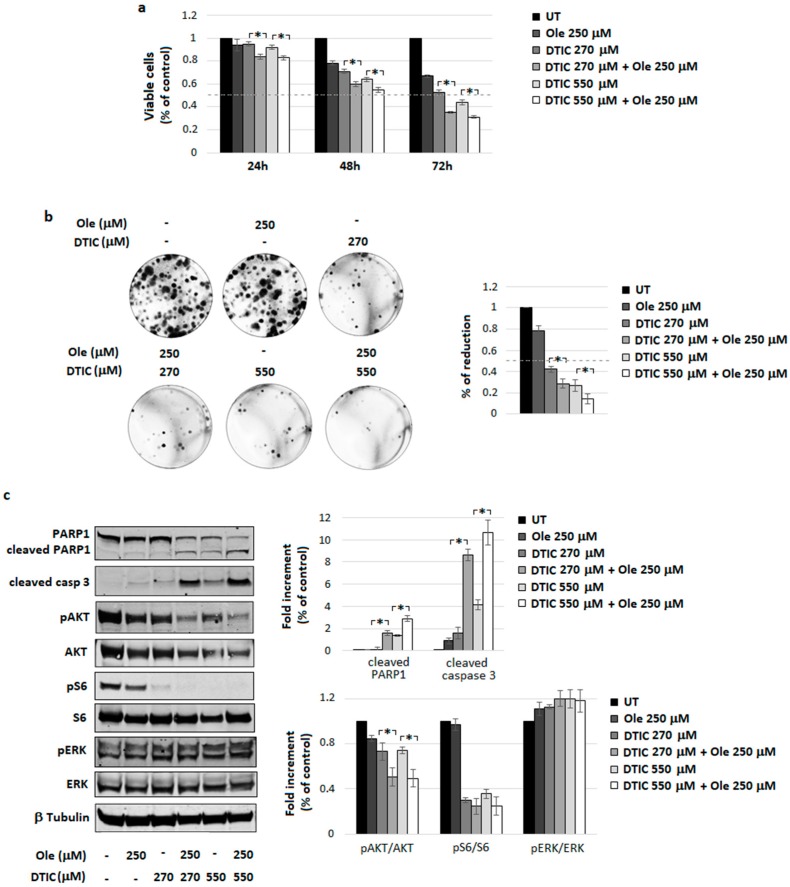
Ole-Dacarbazine (DTIC) efficacy on A375 melanoma cells. (**a**) Dose–time response evaluated via MTT assay. (**b**) (**Left**) Colony Forming Units (CFU) assay of alive cells selected using a trypan blue exclusion test after the treatment with 250 µM (≈125 µg/mL) Ole and/or 270 and 540 µM DTIC (≈50 and 100 µg/mL) for 72 h. (**Right**) Quantification data of colony numbers compared to UT. (**c**) (**Left**) Representative Western blot of PARP1, cleaved PARP1, cleaved caspase 3, pAKT, AKT, pS6, S6, pERK, and ERK after a 250 µM Ole treatment and/or 270 and 540 µM DTIC for 48 h. (**Right**) Densitometric quantification of the cleaved PARP1, cleaved caspase 3, and of the ratio of pERK/ERK, pAKT/AKT, and pS6/S6 relative to β-tubulin expression, expressed as a fold increment (%) compared to UT. * *p* ≤ 0.05 refers to Ole-DTIC treatment vs. DTIC alone. UT = untreated.

The combination of 250 µM Ole plus DTIC (270 and 550 µM corresponding to ≈50 and 100 µg/mL) led to a significant decrease in cell viability with respect to the single treatments, particularly evident at 72 h of incubation (Figure 2a). This finding was confirmed by the level of clonogenicity, suggesting the effectiveness of Ole plus DTIC in reducing the cloning efficiency of melanoma cells (Figure 2b). Furthermore, as shown using Western blot and relative densitometric analysis of Figure 2c, combo treatment elicited a clear expression of cleaved PARP1 and caspase 3, signifying a promotion of apoptosis on treated cells. Ole, by itself, reduced pAKT expression without any modification in pERK levels, but when it was added to DTIC, a significant and more pronounced decrease (by around 30%) in the pAKT/AKT ratio was found (Figure 2c), corroborating the hypothesis of a potential cooperation of Ole with DTIC in the pAKT pathway inhibition.

Overall, Ole cooperates with DTIC in melanoma treatment, possibly participating in the down-regulation of pAKT signaling.

### 4.4. Combination of Ole with Everolimus (RAD001)

**Figure 3:**
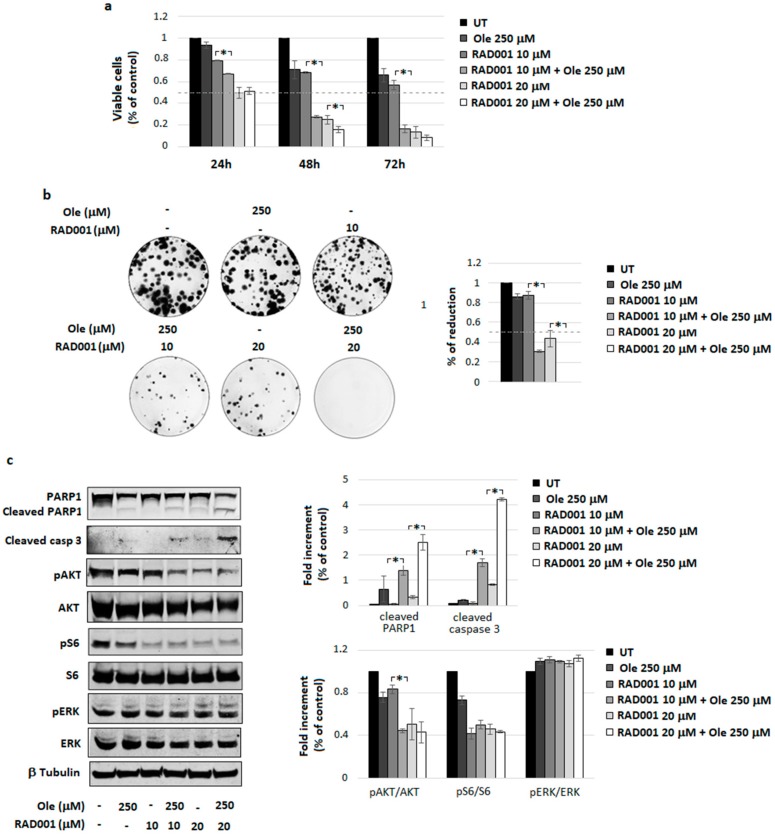
Ole-Everolimus (RAD001) efficacy on A375 melanoma cells. (**a**) Dose–time response evaluated via MTT assay. (**b**) (**Left**) Colonies forming units (CFU) assay of alive cells selected using a trypan blue exclusion test after a 250 µM (≈125 µg/mL) Ole treatment with and/or 10 and 20 µM RAD001 (≈9.5 and 19 µg/mL) for 48 h. (**Right**) Quantification data of the colony numbers compared to UT. (**c**) (**Left**) Representative Western blot of PARP1, cleaved PARP1, cleaved caspase 3, pAKT, AKT, pS6, S6, pERK, and ERK after 250 µM Ole treatment with and/or 10 and 20 µM RAD001 for 24 h. (**Right**) Densitometric quantification of the cleaved PARP1, cleaved caspase 3, and of the ratio of pERK/ERK, pAKT/AKT, and pS6/S6 relative to β-tubulin expression, expressed as a fold increment (%) compared to UT. * *p* ≤ 0.05 refers to Ole-RAD001 treatment vs. RAD001 alone. UT = untreated.

In many cancers, including metastatic melanoma, the PI3K/AKT signal transduction pathway regulates many basic cellular properties. mTOR inhibiting agents, including RAD001, are effective in inhibiting the PI3K/AKT/mTOR pathway, although several reports indicate that mTOR inhibitors have limited single-agent activity against melanoma [34]. Further, our recent research effort demonstrates that RAD001 might overcome PLX4032 resistance acquired via melanoma cells grown under a low extracellular pH [26]. These indications prompted us to study a possible cooperation between Ole and RAD001 and we found that Ole is a potent promoter of RAD001 cytotoxicity (Figure 3a), also confirmed by a significant reduction of cloning efficiency of combo treated melanoma cells compared to single treatments (Figure 3b). The enhanced cell death after combo treatment correlates with an enhanced level of cleaved PARP1 and caspase 3, as shown by the densitometric analysis of the Figure 3c. Further, Ole potentiates the inhibition of pAKT expression exerted via RAD001 at the 10 µM concentration (9.5 µg/mL) by around 35% (Figure 3c).

**Figure 4:**
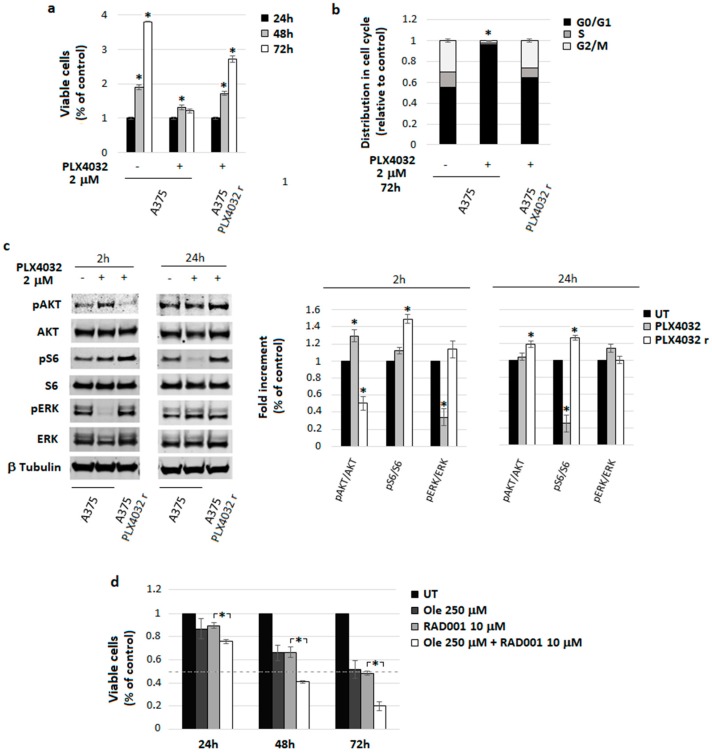
Characterization of A375 melanoma cells resistant to PLX4032 and effect of Ole-RAD001 treatment on these cells. (**a**) Cell viability evaluated via MTT assay. Significance refers to the untreated control. (**b**) Cell cycle distribution analyzed using FACS. * *p* ≤ 0.05 vs. UT. (**c**) Representative Western blot of pAKT, AKT, pS6, S6, pERK, and ERK in cells treated with PLX4032 2 µM (≈980 ng/mL) for 2 or 24 h. (**Right**) Densitometric quantification of the ratio of pERK/ERK, pAKT/AKT, and pS6/S6 relative to β-Tubulin expression. * *p* ≤ 0.05 refers to UT. (**d**) Dose–time response evaluated on A375 cells that were PLX4032-resistant via MTT assay. * *p* ≤ 0.05 refers to Ole-RAD001 treatment vs. RAD001 alone. UT = untreated.

In addition, Ole potentiation of RAD001 treatment might be useful to overcome PLX4032 resistance. In order to confirm this phenomenon, we used A375 PLX4032-resistant melanoma cells, established by Dr. Poliseno [27], during a long period of exposure of A375 cells to PLX4032. These resistant cells grow and proliferate in the presence of PLX4032 in a similar way to control cells (Figure 4a). Further, in contrast with the parental A375 cells, which stopped proliferating and accumulate in the G1 phase of the cell cycle following exposure to PLX4032, resistant cells in the presence of the same amount of drug did not show any modification in cell cycle distribution (Figure 4b). Considering signaling pathways, we found that, resistant cells expressed a higher level of AKT/S6 pathway and an unchanged level of pERK differently from PLX4032-treated cells, which instead underwent a reduction of pERK (at 2 h of treatment) and pS6 (at 24 h of treatment), as shown by the densitometric analysis of the Figure 4c. When we determined the capacity of RAD001-Ole to affect resistant cells, we observed a higher percentage of dead cells with respect to that found after the treatment with RAD001 alone (Figure 4d), suggesting that the special combination of Ole plus RAD001 could represent a new strategy to also treat chronic addicted PLX4032-resistant melanoma cells.

### 4.5. A Freshly Prepared Olive Leaf Extract Potentiates RAD001 Treatment on BRAF Melanoma Cells

This final investigation discloses the possibility that a freshly prepared olive leaf extract enriched in an equimolar concentration of Ole is endowed with the same or greater ability to potentiate drug efficacy. Supplement Figure S3 shows the dry extract composition of green leaves extract, enriched in Ole, with a final concentration of 51.99% *p*/*p* (48.56% of secoiridoids (Oleuropein glucoside and Oleuropein aglycone), 0.92% hydroxytyrosol, tyrosol and derivatives, 1.93% of flavonoids, 0.57% of verbascoside and derivatives), while Supplement Figure S4 shows the quali-quantitative analyses of the solution obtained using Olea powder extract after the solubilization of 56 mg of powder in 5 mL of physiological solution and used for the test in vitro.

**Figure 5:**
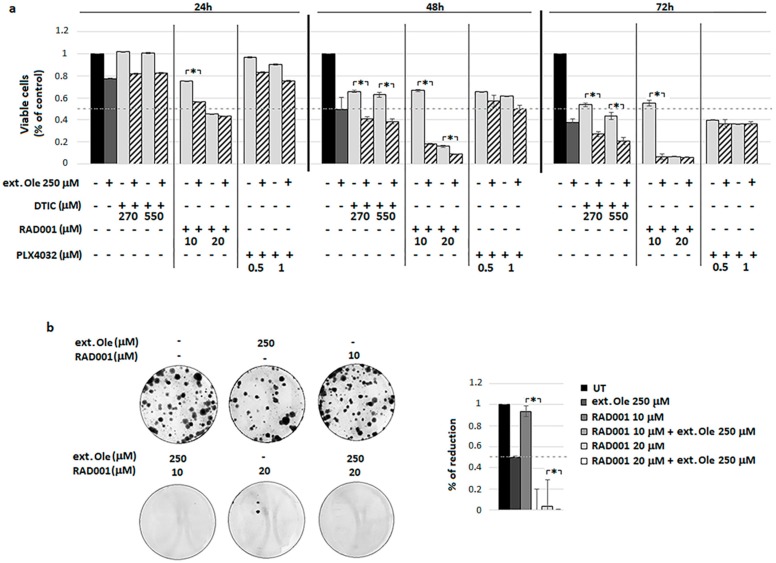
Effects of Ole-enriched leaf extract alone or in combination with DTIC, RAD001, or PLX4032 on A375 melanoma cells. (**a**) Dose–time response evaluated via MTT assay. * *p* ≤ 0.05 refers to OleRAD001/DTIC treatment vs. RAD001/DTIC alone. (**b**) (**Left**) Colonies forming units (CFU) assay of alive cells selected using a trypan blue exclusion test after Ole-enriched leaf extract treatment with and/or RAD001 for 24 h. (**Right**) Quantification data of colony numbers with respect to UT. * *p* ≤ 0.05 refers to Ole-DTIC or Ole-RAD001 treatment compared to DTIC or RAD001 alone. UT = untreated.

The extract enriched in an equimolar concentration of Ole was more effective to potentiate DTIC and especially RAD001 cytotoxicity (Figure 5a) compared to Ole alone (Figure 2a and Figure 3a). As reported in Figure 5, the best combination in inducing cell death on BRAF A375 melanoma cells was represented by the Ole-enriched leaf extract, again, with RAD001, as shown via MTT assay and using the clonogenic assay (Figure 5a,b). This finding confirms previous results, adding a greater potential translation impact to the clinic.

## 5. Discussion

The focus for cancer treatment has been shifted toward strategies of complementary therapy that are able to overcome the limitation of a single-agent treatment. Rational combination approaches are strongly preferred in order to improve the overall patient progression-free survival, overcome or delay the development of drug resistance, and reduce the incidence of side effects. This is of special importance in the treatment of melanoma, particularly of the advanced metastatic form, often resistant to most of the current drugs used in the clinic. Further, due to different transcription pathway activation in melanoma cells, several other mechanisms of resistance to BRAF inhibition have been identified. Melanoma tumors bearing wild-type BRAF are intrinsically resistant to PLX4032 or Dabrafenib. Targeting mitogen-activated protein kinase kinase (MEK) was considered a potential mechanism to overcome BRAF resistance [35], although the most favorable treatment schedule and sequence is still to be defined. Indeed, multiple levels of cross-talk among mitogen-activated protein kinase (MAPK) and PI3K/AKT pathways and the possibility that ERK can be phosphorylated by the pAKT pathway are demonstrated [36,37]. Thus, it is required to also inhibit the AKT pathway in melanoma cells resistant to MAPK inhibitors [38]. The activation of the PI3K/AKT/mTOR pathway represents one of the major mechanisms of acquired resistance to both targeted BRAF inhibitors and DTIC [39,40]. We have found that BRAF melanoma cells exposed to a low extracellular pH medium acquired a resistance to both BRAF and MEK inhibitors but were still sensitive to the inhibition of the AKT/mTOR pathway induced by RAD001 [26]. It is possible, on the other hand, that during a prolonged mTOR inhibition, PI3K would be able to promote an MAPK pathway through RAS activation. Overall, from these findings emerge the need to use a treatment involving the simultaneous inhibition of the MAPK and AKT pathways in order to have a better drug efficiency and reduce drug resistance.

Although this anti-cancer approach appeared very promising, it had not been as successful as once believed. Indeed, most combined therapies are based on the combination of toxic compounds, leading to toxicity and unexpected side effects.

Given our previous evidence for the protective role of Ole in neurodegenerative and cardiovascular diseases [41,42,43,44], we have decided to investigate whether Ole might exert some role in melanoma treatment. The existing studies indicate that Ole expresses a well-demonstrated protective role against many types of cancer [22]. Most of the studies have investigated the anticancer effects of Ole on breast cancer, disclosing that the polyphenol may not only decrease cell viability and proliferation [45] and synergize with Doxorubicin in in vitro and in vivo models [25,46], but also may reverse resistance toward the chemotherapeutic agent, Trastuzumab [47]. In addition, Ole is effective in reducing cell proliferation by increasing apoptosis in human colorectal cancer cells [48] and is able to sensitize the Doxorubicin-mediated killing of prostate cancer cells [24]. Ole also reduces the cell viability of hepatocarcinoma, pancreatic, thyroid, neuroblastoma, mesothelioma, and glioblastoma cancer cells [22]. Still to be clarified are the effects of Ole on BRAF human melanoma cells. Here, we demonstrate for the first time that Ole treatment represents a new non-toxic anti-cancer agent against BRAF melanoma cells as a suitable promoter of two major agents used in BRAF-resistant melanoma cells, such as RAD001 and DTIC. In addition, the particular approach of Ole to potentiate RAD001 was found appropriate to overcome PLX4032 resistance as demonstrated by the use of special PLX4032-resistant A375 melanoma cells developed in cultures. This finding discloses a complementary approach to the therapy of BRAF inhibitor-resistant melanoma that harbors hyperactivation of AKT. Until now, only one study reported the reversing effect of Ole on chemotherapy-induced resistance [47]. Thus, these findings open up the chance to use Ole as a therapeutic molecule to improve the anticancer effects of current chemotherapeutics, due to its low toxicity in normal cells as previously reported [49,50,51].

A375 melanoma cells, used in our study, like most of human solid cancers (prostate, breast, and colon cancer), express the glucose transporter proteins GLUT1 and GLUT3 mRNA and protein, which may likely promote Ole uptake. Hamdi et al. [49] found that the antiproliferative activity of Ole in normal fibroblasts was reduced by removing the glucose moiety by β-glycosidase. Furthermore D-glucose and Ole compete for the GLUTs, as it was demonstrated by the co-incubation of human melanoma cells with an excess of D-glucose, so it is possible that GLUTs are involved in the transportation of Ole into cancer cells. In this study we found that Ole was present in the cytoplasm of A375 cells after only 15 min of incubation (Figure S1b), suggesting a fast uptake of Ole inside the cells, probably due to their higher level of GLUTs compared to the normal ones. This is a very important aspect that we can exploit in thinking of an Ole topical application directly on tumor cells. However, we did not exclude the possibility that Ole could enter into the cells using other routes, in particular, in areas of inflammatory reactions where several mediators are active.

Interestingly, in a very short time (48 h) Ole was able to induce a clear and significant induction of melanoma cell death, confirmed by an enhancement of markers of apoptosis. Ole affected the viability of melanoma cells, probably through the inhibition of phosphorylation of AKT and the S6 pathway. This finding is in accordance with Liu’s observation [52], which indicates that inhibition of AKT is the mechanism underlying the pro-apoptotic and anti-invasive process promoted by Ole in glioma cells and with Yan’s indication [53] about the induction of apoptosis by Ole through the PI3K/AKT pathway in HepG2 human hepatoma cell line.

Thus, we proceeded to investigate whether Ole blocking the AKT pathway may act in cooperation with the current chemotherapy treatments and allow for a decrease the doses that often lead to toxicity and severe side effects. We found that Ole potentiates the cytotoxic effect of DTIC, reducing the effective dose by 50%; this might be related to an enhanced reduction in AKT phosphorylation. In parallel experiments, Ole was also able to potentiate an mTOR inhibitor, such as RAD001, which is also in PLX4032-resistant melanoma cells. In the same way, Ole reduces the effective dose of RAD001 by 50% and this effect was linked to pAKT abrogation. Thus, Ole, by affecting the PI3K/AKT/mTOR pathway, might represent a new non-toxic agent of interest in the treatment of advanced melanoma. It is likely that Ole might also boost up the inhibition of mTOR through the AMPK/mTOR pathway, which in this study was not investigated, but that was suggested by our previous finding and by other authors [43,54]. In addition, Ole may inhibit tumor angiogenesis and in vivo tumor growth, as recently found by the studies of Song et al. [55] and Samara et al. [56].

Although the in vitro studies are very promising, they do not consider Ole metabolism and bioavailability, such that the in vitro used concentrations, despite being in accordance with literature [25,57,58,59,60], could seem far greater than those that could be realistically achieved in in vivo models.

Ole inevitably undergoes a metabolic process in in vivo models, and after ingestion, its metabolites are rapidly detected in plasma at different concentrations depending on gender [61]; however, in the clinic, most agents are typically given via repeated administration that may lead to accumulation [62], and this is quite close to the high doses used in in vitro experiments.

Furthermore our results suggest that an easily available product as an olive leaf extract enriched in Ole could be even more effective than Ole alone, probably because of the co-presence of other polyphenols among HT, which many authors have suggested to be the real active Ole metabolic product [23,31,32]. Therefore, olive leaf extracts seem to have a powerful anticancer property as Samet I. et al. [63] and Mijatovic SA et al. [64] have also shown with regard to human chronic myelogenous leukemia K562 and melanoma cells, respectively, and their mixed phenolic composition with an enrichment in Ole could be useful to decrease the in vitro doses in order to obtain the same effect of Ole administration alone at higher concentration.

Nevertheless, polyphenol bioavailability is still a big drawback that many studies try to overcome through different approaches including the construction of granules containing probiotics and *Olea europaea* extract in order to increase polyphenols bioavailability [65], and the synthesis of more active Ole analogs with various chemical properties [56].

In conclusion, Ole, and even more so olive leaf extracts, exhibited a promising potential as adjuvant in conventional anticancer therapies. Furthermore, it may reverse the drug resistance of cancer cells to chemotherapeutics and reduce adverse effects of conventional therapies on nontarget cells.

The limited in vivo animal studies, as recently summarized by two recent reviews [22,66], and the paucity of in human studies, in particularly randomized controlled clinical trials, still represents the major drawback. Therefore, preclinical evidence needs to be substantiated by an evidence-based approach to determine the effective dose and best route of Ole administration on the basis of its bioavailability [67] and any side effects related to chronic administration.

## Supplementary Materials

The following are available online at http://www.mdpi.com/2072-6643/10/12/1950/s1, Figure S1: Cell viability of A375, WM266-4 and M21 after Ole glucoside (Ole) treatment, Figure S2: Effects of Ole on A375 melanoma cells, Figure S3: Quali-quantitative data of dry extract powder obtained by Olea green leaves extract, Figure S4: Quali-quantitative data of solution used for the test in vitro.

## References

[1] Tsao H., Chin L., Garraway L.A., Fisher D.E. (2012). Melanoma: From mutations to medicine. Genes Dev..

[2] Davies H., Bignell G.R., Cox C., Stephens P., Edkins S., Clegg S., Teague J., Woffendin H., Garnett M.J., Bottomley W. (2002). Mutations of the BRAF gene in human cancer. Nature.

[3] Garnett M.J., Marais R. (2004). Guilty as charged: B-RAF is a human oncogene. Cancer Cell.

[4] Houben R., Becker J.C., Kappel A., Terheyden P., Bröcker E.-B., Goetz R., Rapp U.R. (2004). Constitutive activation of the Ras-Raf signaling pathway in metastatic melanoma is associated with poor prognosis. J. Carcinog..

[5] Wan P.T.C., Garnett M.J., Roe S.M., Lee S., Niculescu-Duvaz D., Good V.M., Jones C.M., Marshall C.J., Springer C.J., Barford D. (2004). Mechanism of activation of the RAF-ERK signaling pathway by oncogenic mutations of B-RAF. Cell.

[6] Wellbrock C., Ogilvie L., Hedley D., Karasarides M., Martin J., Niculescu-Duvaz D., Springer C.J., Marais R. (2004). V599EB-RAF is an oncogene in melanocytes. Cancer Res..

[7] Chin L., Garraway L.A., Fisher D.E. (2006). Malignant melanoma: Genetics and therapeutics in the genomic era. Genes Dev..

[8] Flaherty K.T., Robert C., Hersey P., Nathan P., Garbe C., Milhem M., Demidov L.V., Hassel J.C., Rutkowski P., Mohr P. (2012). Improved survival with MEK inhibition in BRAF-mutated melanoma. N. Engl. J. Med..

[9] Davies M.A. (2012). The role of the PI3K-AKT pathway in melanoma. Cancer J..

[10] Valls-Pedret C., Lamuela-Raventós R.M., Medina-Remón A., Quintana M., Corella D., Pintó X., Martínez-González M.Á., Estruch R., Ros E. (2012). Polyphenol-rich foods in the Mediterranean diet are associated with better cognitive function in elderly subjects at high cardiovascular risk. J. Alzheimer’s Dis..

[11] Santoro A., Pini E., Scurti M., Palmas G., Berendsen A., Brzozowska A., Pietruszka B., Szczecinska A., Cano N., Meunier N. (2014). Combating inflammaging through a Mediterranean whole diet approach: The NU-AGE project’s conceptual framework and design. Mech. Ageing Dev..

[12] van den Brandt P.A., Schulpen M. (2017). Mediterranean diet adherence and risk of postmenopausal breast cancer: Results of a cohort study and meta-analysis. Int. J. Cancer.

[13] Sofi F., Abbate R., Gensini G.F., Casini A. (2010). Accruing evidence on benefits of adherence to the Mediterranean diet on health: An updated systematic review and meta-analysis. Am. J. Clin. Nutr..

[14] Ruiz-Canela M., Martínez-González M.A. (2011). Olive oil in the primary prevention of cardiovascular disease. Maturitas.

[15] Visioli F., Franco M., Toledo E., Luchsinger J., Willett W.C., Hu F.B., Martinez-Gonzalez M.A. (2018). Olive oil and prevention of chronic diseases: Summary of an International conference. Nutr. Metab. Cardiovasc. Dis..

[16] Oliveras-López M.-J., Molina J.J.M., Mir M.V., Rey E.F., Martín F., de la Serrana H.L.-G. (2013). Extra virgin olive oil (EVOO) consumption and antioxidant status in healthy institutionalized elderly humans. Arch. Gerontol. Geriatr..

[17] Cicerale S., Lucas L.J., Keast R.S.J. (2012). Antimicrobial, antioxidant and anti-inflammatory phenolic activities in extra virgin olive oil. Curr. Opin. Biotechnol..

[18] Barbaro B., Toietta G., Maggio R., Arciello M., Tarocchi M., Galli A., Balsano C. (2014). Effects of the olive-derived polyphenol oleuropein on human health. Int. J. Mol. Sci..

[19] Crespo M.C., Tomé-Carneiro J., Dávalos A., Visioli F. (2018). Pharma-Nutritional Properties of Olive Oil Phenols. Transfer of New Findings to Human Nutrition. Foods.

[20] Hassen I., Casabianca H., Hosni K. (2015). Biological activities of the natural antioxidant oleuropein: Exceeding the expectation—A mini-review. J. Funct. Foods.

[21] Angeloni C., Malaguti M., Barbalace M.C., Hrelia S. (2017). Bioactivity of Olive Oil Phenols in Neuroprotection. Int. J. Mol. Sci..

[22] Shamshoum H., Vlavcheski F., Tsiani E. (2017). Anticancer effects of oleuropein. Biofactors.

[23] Imran M., Nadeem M., Gilani S.A., Khan S., Sajid M.W., Amir R.M. (2018). Antitumor Perspectives of Oleuropein and Its Metabolite Hydroxytyrosol: Recent Updates. J. Food Sci..

[24] Papachristodoulou A., Tsoukala M., Benaki D., Kostidis S., Gioti K., Aligiannis N., Pratsinis H., Kletsas D., Skaltsounis A.-L., Mikros E. (2018). Oleuropein is a Powerful Sensitizer of Doxorubicin-mediated Killing of Prostate Cancer Cells and Exerts Its Action via Induction of Autophagy. J. Cancer Res. Treat..

[25] Choupani J., Alivand M.R., Derakhshan S.M., Zaeifizadeh M., Khaniani M.S. (2018). Oleuropein inhibits migration ability through suppression of epithelial-mesenchymal transition and synergistically enhances doxorubicin-mediated apoptosis in MCF-7 cells. J. Cell. Physiol..

[26] Ruzzolini J., Peppicelli S., Andreucci E., Bianchini F., Margheri F., Laurenzana A., Fibbi G., Pimpinelli N., Calorini L. (2017). Everolimus selectively targets vemurafenib resistant BRAFV600E melanoma cells adapted to low pH. Cancer Lett..

[27] Vitiello M., Tuccoli A., D’Aurizio R., Sarti S., Giannecchini L., Lubrano S., Marranci A., Evangelista M., Peppicelli S., Ippolito C. (2017). Context-dependent miR-204 and miR-211 affect the biological properties of amelanotic and melanotic melanoma cells. Oncotarget.

[28] Pagnini I., Simonini G., Cavalli L., la Marca G., Iuliano A., Brandi M., Bellisai F., Frediani B., Galeazzi M., Cantarini L. (2014). Bone status of children born from mothers with autoimmune diseases treated during pregnancy with prednisone and/or low molecular weight heparin. Pediatr. Rheumatol..

[29] Romani A., Mulas S., Heimler D. (2017). Polyphenols and secoiridoids in raw material (*Olea europaea* L. leaves) and commercial food supplements. Eur. Food Res. Technol..

[30] Romani A., Scardigli A., Pinelli P. (2017). An environmentally friendly process for the production of extracts rich in phenolic antioxidants from *Olea europaea* L. and *Cynara scolymus* L. matrices. Eur. Food Res. Technol..

[31] Robles-Almazan M., Pulido-Moran M., Moreno-Fernandez J., Ramirez-Tortosa C., Rodriguez-Garcia C., Quiles J.L., Ramirez-Tortosa M. (2018). Hydroxytyrosol: Bioavailability, toxicity, and clinical applications. Food Res. Int..

[32] Carrera-González M.P., Ramírez-Expósito M.J., Mayas M.D., Martínez-Martos J.M. (2013). Protective role of oleuropein and its metabolite hydroxytyrosol on cancer. Trends Food Sci. Technol..

[33] Mouawad R., Sebert M., Michels J., Bloch J., Spano J.-P., Khayat D. (2010). Treatment for metastatic malignant melanoma: Old drugs and new strategies. Crit. Rev. Oncol. Hematol..

[34] Yap T.A., Yan L., Patnaik A., Fearen I., Olmos D., Papadopoulos K., Baird R.D., Delgado L., Taylor A., Lupinacci L. (2011). First-in-man clinical trial of the oral pan-AKT inhibitor MK-2206 in patients with advanced solid tumors. J. Clin. Oncol..

[35] Lito P., Pratilas C.A., Joseph E.W., Tadi M., Halilovic E., Zubrowski M., Huang A., Wong W.L., Callahan M.K., Merghoub T. (2012). Relief of profound feedback inhibition of mitogenic signaling by RAF inhibitors attenuates their activity in BRAFV600E melanomas. Cancer Cell.

[36] Grammer T.C., Blenis J. (1997). Evidence for MEK-independent pathways regulating the prolonged activation of the ERK-MAP kinases. Oncogene.

[37] Jiang C.C., Lai F., Thorne R.F., Yang F., Liu H., Hersey P., Zhang X.D. (2011). MEK-independent survival of B-RAFV600E melanoma cells selected for resistance to apoptosis induced by the RAF inhibitor PLX4720. Clin. Cancer Res..

[38] Vivanco I., Sawyers C.L. (2002). The phosphatidylinositol 3-Kinase AKT pathway in human cancer. Nat. Rev. Cancer.

[39] Perna D., Karreth F.A., Rust A.G., Perez-Mancera P.A., Rashid M., Iorio F., Alifrangis C., Arends M.J., Bosenberg M.W., Bollag G. (2015). BRAF inhibitor resistance mediated by the AKT pathway in an oncogenic BRAF mouse melanoma model. Proc. Natl. Acad. Sci. USA.

[40] Chi M., Ye Y., Zhang X.D., Chen J. (2014). Insulin induces drug resistance in melanoma through activation of the PI3K/Akt pathway. Drug Des. Dev. Ther..

[41] Grossi C., Rigacci S., Ambrosini S., Ed Dami T., Luccarini I., Traini C., Failli P., Berti A., Casamenti F., Stefani M. (2013). The polyphenol oleuropein aglycone protects TgCRND8 mice against Aß plaque pathology. PLoS ONE.

[42] Miceli C., Santin Y., Manzella N., Coppini R., Berti A., Stefani M., Parini A., Mialet-Perez J., Nediani C. (2018). Oleuropein Aglycone Protects against MAO-A-Induced Autophagy Impairment and Cardiomyocyte Death through Activation of TFEB. Oxidative Med. Cell. Longev..

[43] Rigacci S., Miceli C., Nediani C., Berti A., Cascella R., Pantano D., Nardiello P., Luccarini I., Casamenti F., Stefani M. (2015). Oleuropein aglycone induces autophagy via the AMPK/mTOR signalling pathway: A mechanistic insight. Oncotarget.

[44] Luccarini I., Pantano D., Nardiello P., Cavone L., Lapucci A., Miceli C., Nediani C., Berti A., Stefani M., Casamenti F. (2016). The Polyphenol Oleuropein Aglycone Modulates the PARP1-SIRT1 Interplay: An In Vitro and In Vivo Study. J. Alzheimer’s Dis..

[45] Liman R., Çoban F., Ciğerci I., Bulduk İ., Bozkurt S. (2017). Antiangiogenic and Apoptotic Effects of Oleuropein on Breast Cancer Cells. Br. J. Pharm. Res..

[46] Elamin M.H., Elmahi A.B., Daghestani M.H., Al-Olayan E.M., Al-Ajmi R.A., Alkhuriji A.F., Hamed S.S., Elkhadragy M.F. (2017). Synergistic Anti-Breast-Cancer Effects of Combined Treatment with Oleuropein and Doxorubicin In Vivo. Altern. Ther. Health Med..

[47] Menendez J.A., Vazquez-Martin A., Colomer R., Brunet J., Carrasco-Pancorbo A., Garcia-Villalba R., Fernandez-Gutierrez A., Segura-Carretero A. (2007). Olive oil’s bitter principle reverses acquired autoresistance to trastuzumab (HerceptinTM) in HER2-overexpressing breast cancer cells. BMC Cancer.

[48] Notarnicola M., Pisanti S., Tutino V., Bocale D., Rotelli M.T., Gentile A., Memeo V., Bifulco M., Perri E., Caruso M.G. (2011). Effects of olive oil polyphenols on fatty acid synthase gene expression and activity in human colorectal cancer cells. Genes Nutr..

[49] Hamdi H.K., Castellon R. (2005). Oleuropein, a non-toxic olive iridoid, is an anti-tumor agent and cytoskeleton disruptor. Biochem. Biophys. Res. Commun..

[50] Goldsmith C., Bond D., Jankowski H., Weidenhofer J., Stathopoulos C., Roach P., Scarlett C. (2018). The Olive Biophenols Oleuropein and Hydroxytyrosol Selectively Reduce Proliferation, Influence the Cell Cycle, and Induce Apoptosis in Pancreatic Cancer Cells. Int. J. Mol. Sci..

[51] Haris Omar S. (2010). Oleuropein in Olive and its Pharmacological Effects. Sci. Pharm..

[52] Liu M., Wang J., Huang B., Chen A., Li X. (2016). Oleuropein inhibits the proliferation and invasion of glioma cells via suppression of the AKT signaling pathway. Oncol. Rep..

[53] Yan C.-M., Chai E.-Q., Cai H.-Y., Miao G.-Y., Ma W. (2015). Oleuropein induces apoptosis via activation of caspases and suppression of phosphatidylinositol 3-kinase/protein kinase B pathway in HepG2 human hepatoma cell line. Mol. Med. Rep..

[54] Pazoki-Toroudi H., Amani H., Ajami M., Nabavi S.F., Braidy N., Kasi P.D., Nabavi S.M. (2016). Targeting mTOR signaling by polyphenols: A new therapeutic target for ageing. Ageing Res. Rev..

[55] Song H., Lim D.Y., Jung J.I., Cho H.J., Park S.Y., Kwon G.T., Kang Y.-H., Lee K.W., Choi M.-S., Park J.H.Y. (2017). Dietary oleuropein inhibits tumor angiogenesis and lymphangiogenesis in the B16F10 melanoma allograft model: A mechanism for the suppression of high-fat diet-induced solid tumor growth and lymph node metastasis. Oncotarget.

[56] Samara P., Christoforidou N., Lemus C., Argyropoulou A., Ioannou K., Vougogiannopoulou K., Aligiannis N., Paronis E., Gaboriaud-Kolar N., Tsitsilonis O. (2017). New semi-synthetic analogs of oleuropein show improved anticancer activity in vitro and in vivo. Eur. J. Med. Chem..

[57] Vanella L. (2012). Antiproliferative effect of oleuropein in prostate cell lines. Int. J. Oncol..

[58] Han J., Talorete T.P.N., Yamada P., Isoda H. (2009). Anti-proliferative and apoptotic effects of oleuropein and hydroxytyrosol on human breast cancer MCF-7 cells. Cytotechnology.

[59] Cárdeno A., Sánchez-Hidalgo M., Rosillo M.A., de la Lastra C.A. (2013). Oleuropein, a Secoiridoid Derived from Olive Tree, Inhibits the Proliferation of Human Colorectal Cancer Cell Through Downregulation of HIF-1α. Nutr. Cancer.

[60] Yao J., Wu J., Yang X., Yang J., Zhang Y., Du L. (2014). Oleuropein induced apoptosis in HeLa cells via a mitochondrial apoptotic cascade associated with activation of the c-Jun NH2-terminal kinase. J. Pharmacol. Sci..

[61] de Bock M., Thorstensen E.B., Derraik J.G.B., Henderson H.V., Hofman P.L., Cutfield W.S. (2013). Human absorption and metabolism of oleuropein and hydroxytyrosol ingested as olive (*Olea europaea* L.) leaf extract. Mol. Nutr. Food Res..

[62] Liston D.R., Davis M. (2017). Clinically Relevant Concentrations of Anticancer Drugs: A Guide for Nonclinical Studies. Clin. Cancer Res..

[63] Samet I., Han J., Jlaiel L., Sayadi S., Isoda H. (2014). Olive (*Olea europaea*) Leaf Extract Induces Apoptosis and Monocyte/Macrophage Differentiation in Human Chronic Myelogenous Leukemia K562 Cells: Insight into the Underlying Mechanism. Oxidative Med. Cell. Longev..

[64] Mijatovic S.A., Timotijevic G.S., Miljkovic D.M., Radovic J.M., Maksimovic-Ivanic D.D., Dekanski D.P., Stosic-Grujicic S.D. (2011). Multiple antimelanoma potential of dry olive leaf extract. Int. J. Cancer.

[65] Aponte M., Ungaro F., d’Angelo I., De Caro C., Russo R., Blaiotta G., Dal Piaz F., Calignano A., Miro A. (2018). Improving in vivo conversion of oleuropein into hydroxytyrosol by oral granules containing probiotic Lactobacillus plantarum 299v and an *Olea europaea* standardized extract. Int. J. Pharm..

[66] Fabiani R. (2016). Anti-cancer properties of olive oil secoiridoid phenols: A systematic review of in vivo studies. Food Funct..

[67] Tresserra-Rimbau A., Lamuela-Raventos R.M., Moreno J.J. (2018). Polyphenols, food and pharma. Current knowledge and directions for future research. Biochem. Pharmacol..

