# Supplementary material for: Oleuropein, the Main Polyphenol of Olea europaea Leaf Extract, Has an Anti-Cancer Effect on Human BRAF Melanoma Cells and Potentiates the Cytotoxicity of Current Chemotherapies
Source: Nutrients. 2018 Dec 8;10(12):1950. doi: 10.3390/nu10121950 (PMC6316801; doi:10.3390/nu10121950)
Supplement: Supplementary file 1 [file nutrients-10-01950-s001.pdf]

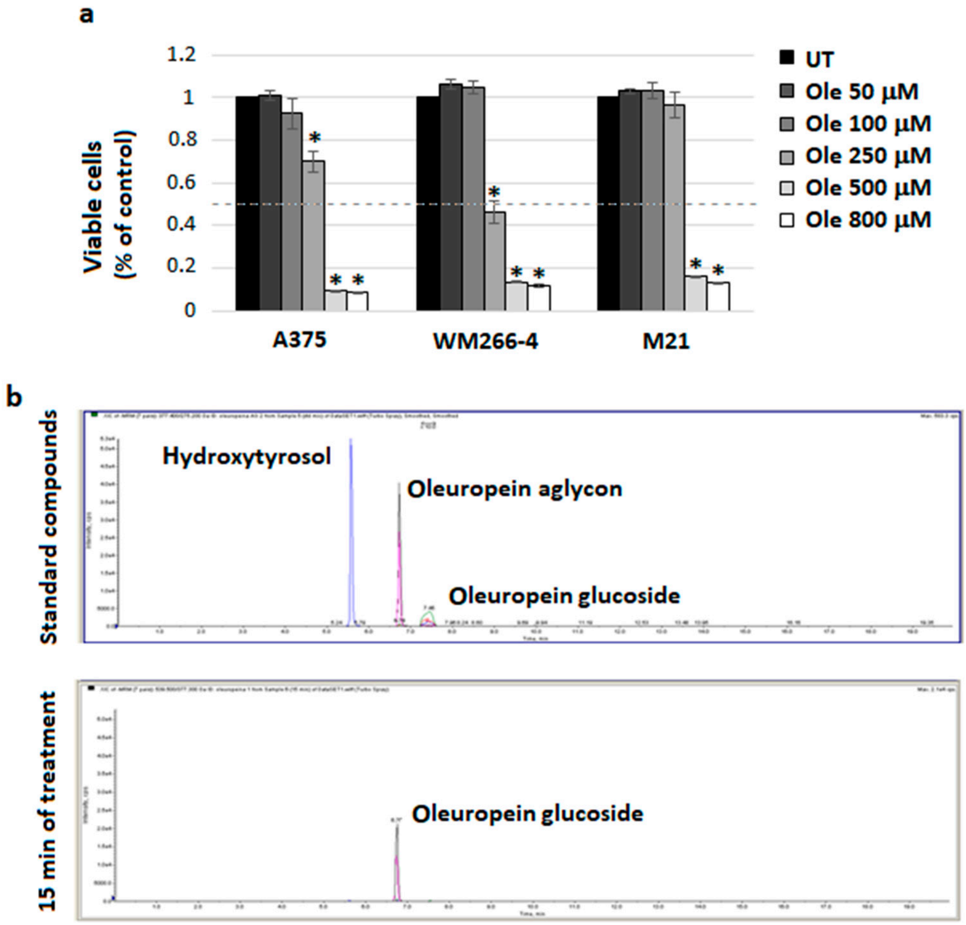

Supplement Figure S1. Cell viability of A375, WM266-4 and M21 after Ole glucoside (Ole) treatment. (**a**) Dose response of cell viability assessed by MTT assay after 72 h of Ole treatment; (**b**) Detection of Ole and its metabolites in A375 melanoma cells after 15 minutes of Ole treatment by Mass Spectrometry. *$p \leq 0.05$ vs UT (=untreated).

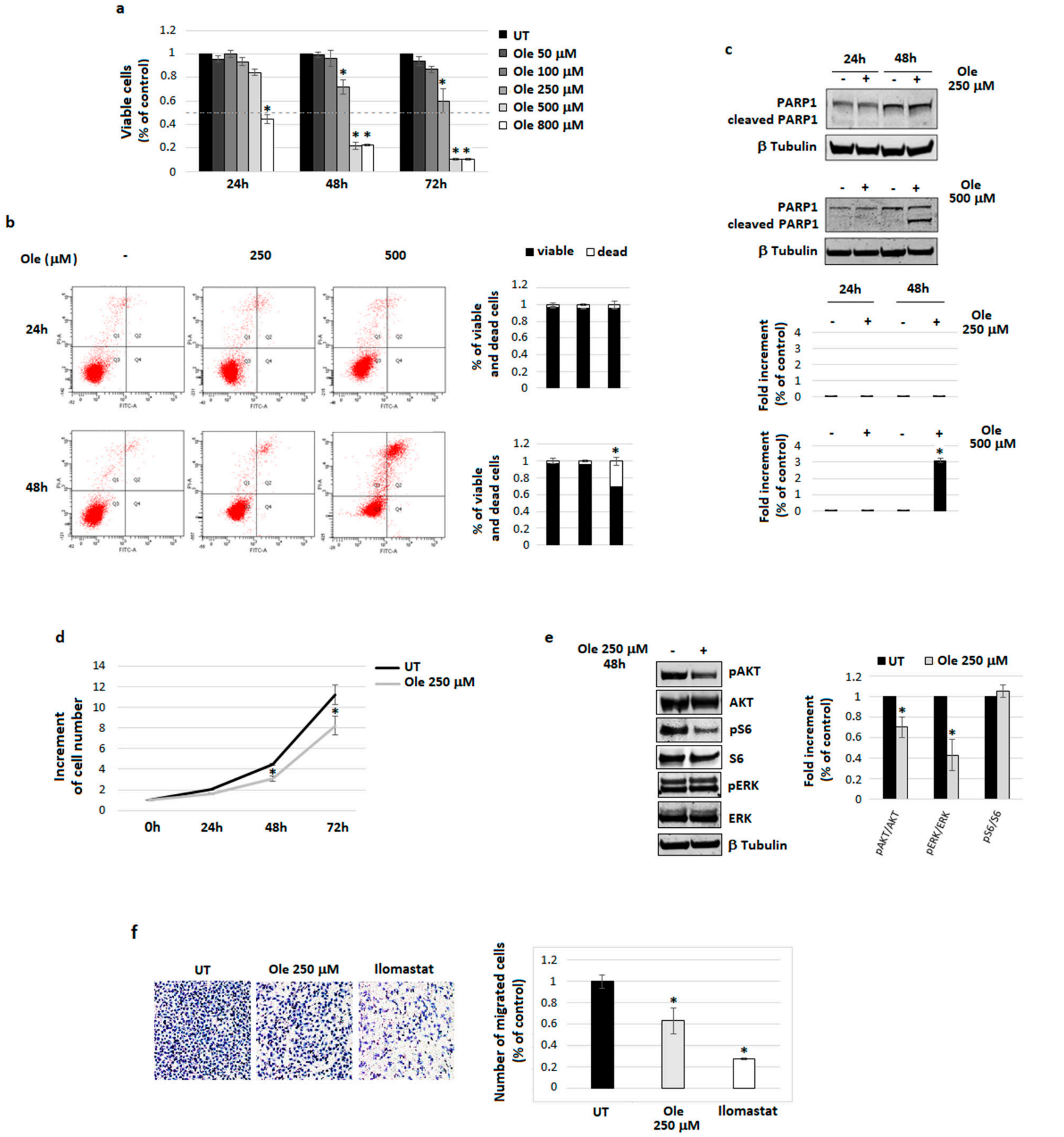

Supplement Figure S2. Effects of Ole on A375 melanoma cells.

(**a**) Dose-time response evaluated by MTT assay; (**b**) (Left) Melanoma cells apoptosis after 250 (≈125 μg/mL) or 500 μM(≈250 μg/mL) Ole treatment for 24 and 48h, analyzed by FACS through cellular incorporation of PI and Annexin V-FITC; (Right) Quantitative data; (**c**) (Upper) Western blot analysis of PARP1 and cleaved PARP1 from cells treated 24 or 48h with 250 or 500 μM Ole; (Lower) Densitometric quantification of cleaved PARP1 protein expression relative to β-Tubulin (**d**) Time-dependent increase of cell number in the presence of 250 μM Ole ; (**e**) Western blot analysis of pAKT, AKT, pS6, S6, pERK and ERK from cells treated with 250 μM Ole for 48h. (Right) Densitometric quantification of the

ratio of pAKT/AKT, pERK/ERK, pS6/S6 relative to β-Tubulin expression; (**f**) Invasiveness of melanoma cells after 250 μM Ole treatment for 24h. Invasive assay was performed using filters coated with matrigel. 25 μM Ilomastat treatment was used as positive control for the inhibition of metalloprotease activity. Migration reduction level was expressed as a percentage of UT. *$p \leq 0.05$ vs UT (=untreated).

	**mg/g dry extract powder**
Hydroxytyrosol	6.59
Tyrosol and derivatives	2.58
Verbascoside and derivatives	5.72
Flavonols	19.23
Oleuropein glucoside	410.27
Oleuropein aglycone	75.48
**Total polyphenols**	**519.87**

Supplement Figure S3. Quali-quantitative data of dry extract powder obtained by Olea green leaves extract.
Data are presented as the mean of three determinations (standard deviation < 3%) and expressed in mg/g dry extract powder.

	**mM extract powder solution**	**% composition**
Hydroxytyrosol	0,523	4.16%
Tyrosol and derivatives	0,345	2.74%
Verbascoside and derivvatives	0,057	0.45 %
Flavonols	0,353	2.81 %
Oleuropein glucoside	8,994	71.47%
Oleuropein	2,312	18.37%
**Total polyphenols**	**12,584**	**100 %**

Supplement Figure S4. Quali-quantitative data of solution used for the test in vitro.
Data are presented as the mean of three determinations (standard deviation < 3%) and expressed as percentage of mg/g dry extract powder.
